# In Vitro and In Vivo Assessment of Metabolic Drug Interaction Potential of Dutasteride with Ketoconazole

**DOI:** 10.3390/pharmaceutics11120673

**Published:** 2019-12-11

**Authors:** Seong-Wook Seo, Jin Woo Park, Dong-Gyun Han, Ji-Min Kim, Sanghyun Kim, Taeuk Park, Kyung-Hwa Kang, Min Hye Yang, In-Soo Yoon

**Affiliations:** 1 College of Pharmacy, Pusan National University, Busan 46241, Korea; sswook@pusan.ac.kr (S.-W.S.); hann9607@pusan.ac.kr (D.-G.H.); jiminkim@pusan.ac.kr (J.-M.K.); 2 Department of Pharmacy, College of Pharmacy, Mokpo National University, Jeonnam 58554, Korea; jwpark@mokpo.ac.kr; 3 Laboratory Animal Center, Daegu-Gyeongbuk Medical Innovation Foundation, Daegu 41061, Korea; shkim@dgmif.re.kr (S.K.); tw3000@dgmif.re.kr (T.P.); 4 Department of Physiology, College of Korean Medicine, Dongeui University, Busan 47227, Korea

**Keywords:** dutasteride, 6β-hydroxy dutasteride, HPLC, fluorescence, ketoconazole, CYP3A

## Abstract

Dutasteride (DUT) is a selective, potent, competitive, and irreversible inhibitor of both type-1 and type-2 5α-reductase (5AR) commonly used in the treatment of benign prostatic hyperplasia and androgenetic alopecia. In the present study, we developed a simple and sensitive high-performance liquid chromatography with fluorescence detection (HPLC-FL) method for simultaneous determination of DUT and its major active metabolite, 6β-hydroxydutasteride (H-DUT). Next, the pharmacokinetic interactions of DUT with ketoconazole (KET), a potent CYP3A inhibitor, were comprehensively investigated. In vivo rat intravenous and oral studies revealed that the pharmacokinetics of DUT and H-DUT were significantly altered by the co-administration of KET. Furthermore, the in vitro microsomal metabolism, blood distribution, and protein-binding studies suggest that the altered pharmacokinetics of DUT could be attributed primarily to the inhibition of the DUT metabolism by KET. To the best of our knowledge, this is the first study to show the drug interaction potential of DUT with azole antifungal drugs including KET, together with a newly developed HPLC-FL method for the simultaneous quantification of DUT and H-DUT.

## 1. Introduction

**Figure 1:**
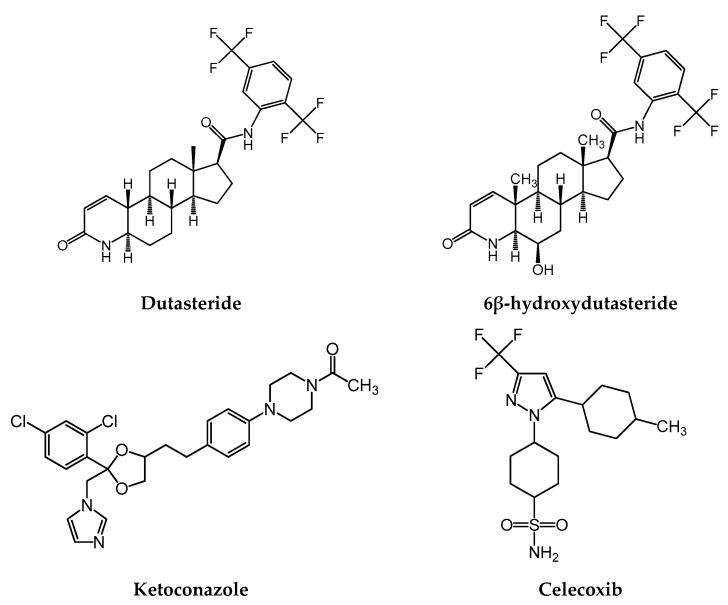
Chemical structures of dutasteride (DUT), 6β-hydroxydutasteride (H-DUT), ketoconazole (KET), and celecoxib (internal standard).

Dutasteride (DUT; Figure 1), marketed under the brand name Avodart (GlaxoSmithKline), is a selective, potent, competitive, and irreversible inhibitor of 5α-reductase (5AR) that catalyzes the intracellular conversion of testosterone to dihydrotestosterone [1]. Dihydrotestosterone is known to be closely associated with benign prostatic hyperplasia (BPH) and androgenetic alopecia [2,3,4]. To date, only two isozymes of 5AR have been identified: type 1, which is primarily found in the liver and skin, and type 2, which is predominantly found in the hair follicles and male genitalia [1,5]. DUT, a dual inhibitor of type-1 and type-2 5AR, was approved by the US Food and Drug Administration (FDA) in 2001 for the treatment of symptomatic benign prostatic hyperplasia [6]. Further, it was approved for androgenic alopecia in Korea and Japan [7]. Previous in vitro studies have shown that compared to finasteride, DUT more potently inhibited type-1 and type-2 5AR by 45- and 2.5-fold, respectively [8,9].

In humans, the oral bioavailability of DUT is approximately 60%, and ranges between 40% and 94%. DUT has a relatively large volume of distribution and high plasma protein binding. The major elimination route of DUT is cytochrome P450 (CYP) 3A4/3A5-mediated hepatic metabolism. Among three major and two minor metabolites, 6β-hydroxydutasteride (H-DUT; Figure 1) exerts 5AR inhibition activity similar to that of DUT [6]. Only trace amounts of unchanged DUT are excreted in the urine [7]. The terminal half-life of DUT after reaching a steady state is approximately 5 weeks [6]. Although the toxicodynamics (relationship between drug concentration and drug-related toxicity) of DUT is currently unknown, the FDA prescription drug label of DUT indicates that metabolic drug interactions may occur when DUT is administered in combination with potent CYP3A inhibitors such as ketoconazole (KET), ritonavir, and troleandomycin [6]. To date, however, this issue has not been adequately addressed by any systematic preclinical and clinical studies.

As shown in Table S1, several methods using liquid chromatography coupled with tandem mass detector (LC-MS/MS) and high-performance liquid chromatography coupled with UV/Vis detector (HPLC-UV) were developed for the quantitation of DUT in rat and human plasma. However, some of these methods were not fully validated, used a large sample volume, and/or had drawbacks associated with sample preparation procedures such as liquid–liquid extraction and solid phase extraction, i.e., high cost, irregular recovery, and/or potential hazards associated with highly volatile solvents. Moreover, LC-MS/MS systems require high instrumentation and maintenance expenses, which may not be affordable for many institutions in resource-limited conditions [10,11]. In this regard, HPLC with fluorescence detection (HPLC-FL) methods can be a good alternative, because they are more accessible and cost effective than LC-MS/MS systems, and they generally offer better sensitivity and selectivity than HPLC-UV systems [12,13]. Additionally, the blood levels of H-DUT, the major active metabolite, need to be monitored together with those of DUT [6]. To the best of our knowledge, there have been no studies showing a validated HPLC-FL method for the quantitative determination of DUT and H-DUT in biological matrices.

In this study, a simple bioanalytical method using the HPLC-FL system was developed for the simultaneous quantitation of DUT and H-DUT in rat plasma and was validated for its original application to in vivo rat pharmacokinetic study on DUT. The potential of the pharmacokinetic drug interaction of DUT with KET (Figure 1), an azole antifungal drug that is a potent CYP3A inhibitor [11,14], was investigated in terms of in vitro rat and human microsomal metabolism, in vitro protein binding, and in vivo rat pharmacokinetics. Approximately 50% of men aged over 50 years have pathological evidence of BPH and this number increases to >80% of men aged over 80 years [15]. Moreover, almost a billion people are estimated to have skin, nail and hair fungal infections, several tens of millions of people have mucosal candidiasis, and more than 150 million people have serious fungal diseases, which are fatal or significantly affect quality of life [16]. To the best of our knowledge, data are not available on the prevalence of fungal infection in patients with BPH, but it cannot exclude the possibility of concurrent use of the two drugs, especially in male elders, when considering the high prevalence rate of each disease. Additionally, KET can serve as a model CYP3A4 inhibitor in this study, which may facilitate further investigation on clinical pharmacokinetic and pharmacodynamic interactions of DUT with other chronic CYP3A4 inhibitors.

## 2. Materials and Methods

### 2.1. Materials

Dutasteride (purity ≥ 99%) was purchased from Carbosynth Korea (Seoul, Korea). H-DUT (purity > 98%) was purchased from Toronto Research Chemicals Inc. (North York, ON, Canada). KET (purity > 98%) and celecoxib (purity > 98%; as an internal standard; Figure 1) were purchased from Tokyo Chemical Industry Co., Ltd. (Tokyo, Japan). Pooled plasma from male Sprague–Dawley rats was purchased from Innovative Research, Inc. (Novi, MI, USA). Nicotinamide adenine dinucleotide phosphate (NADPH), rat liver microsomes (RLM; from male Sprague–Dawley rats), and human liver microsomes (HLM) were purchased from BD-Genetech (Woburn, MA, USA). HPLC-grade acetonitrile (ACN), dimethyl sulfoxide, methanol, and ethanol were purchased from Thermo Fisher Scientific, Inc. (Waltham, MA, USA). Polyethylene glycol 400 was purchased from Sigma-Aldrich Co. (St. Louis, MO, USA).

### 2.2. Spectrofluorometric Evaluation

Fluorescence intensities of DUT and H-DUT (dissolved in methanol at 10 μg/mL) were measured using a spectrofluorometer (2475 FLR Detector; Waters Co., Milford, MA, USA) at various excitation and emission wavelengths. A pair of excitation and emission wavelengths that produced the maximum fluorescence intensity was chosen.

### 2.3. Preparation of Calibration Standards and Quality Control Samples

Stock solutions of DUT and H-DUT (1000 μg/mL) were prepared with dimethyl sulfoxide. The stock solutions were diluted serially using methanol to prepare working standard solutions with concentrations ranging from 1 to 100 μg/mL. To prepare calibration standard samples, blank rat plasma was spiked with each working standard solution, yielding final plasma concentrations of 10, 20, 50, 100, 200, 500, and 1000 ng/mL. The quality control (QC) samples were prepared in the same manner as the calibration standards, yielding final concentrations of 10 ng/mL (lower limit of quantification; LLOQ, which is defined as the lowest quantifiable concentration levels of DUT and H-DUT in calibration curves), 30 (low; LQC), 150 (middle; MQC), and 750 (high; HQC). The plasma sample (100 μL) was deproteinized with 300 μL of ice-cold ACN containing celecoxib as an internal standard (400 ng/mL). After vortex-mixing for 5 min followed by centrifugation at 15,000× *g* for 10 min, 300 μL of the supernatant was transferred to a clean microtube and then allowed to evaporate under a gentle nitrogen gas stream. The resultant dried residue was reconstituted with 50 μL of the mobile phase, and 20 μL aliquot was injected into the HPLC-FL system.

### 2.4. Chromatographic Conditions

A Shimadzu HPLC system combined with fluorescence detector (Shimadzu Co., Kyoto, Japan) was used in this study. Chromatographic separation was conducted at 40 °C using a Kinetex C8 column (250 × 4.6 mm, 5 μm, 100 Å; Phenomenex, Torrance, CA, USA) protected by a C8 guard column (SecurityGuard HPLC Cartridge System, Phenomenex, Torrance, CA, USA). The gradient elution of the mobile phase consisting of pH 6.0 10 mM potassium phosphate buffer (Solvent A) and ACN (Solvent B) was performed at a flow rate of 1 mL/min as follows (solvent A:solvent B, *v/v*): maintained at 58:42 for 12.5 min; ramped from 58:42 to 50:50 for 0.5 min; maintained at 50:50 for 9 min; back to 58:42 for 0.5 min; and maintained for 3.5 min (total run time: 26 min).

### 2.5. Bioanalytical Method Validation

The bioanalytical method proposed herein for the simultaneous determination of DUT and H-DUT was fully validated on the basis of the US FDA guideline [17]. The selectivity of the method was evaluated by comparing chromatograms of the analytes (DUT, H-DUT, and internal standard) in blank rat plasma, blank rat plasma spiked with the analytes, and rat plasma sample obtained from the in vivo rat pharmacokinetic study. Further, the existence of potential interferences at the acquisition windows of the analytes was checked. The linearity of the method was evaluated by adding increasing amounts of DUT and H-DUT to the blank plasma. To construct calibration curves (*n* = 5), the peak area ratios of DUT or H-DUT to the internal standard (*y*-axis) were plotted against the nominal concentration ratios of DUT or H-DUT (10–1000 ng/mL) to the internal standard (400 ng/mL) (*x*-axis), and then, linear regression analysis was performed. The sensitivity was assessed by estimating the LLOQ (signal-to-noise [S/N] ratio > 5). The peaks of the analytes at the LLOQ should be discrete, identifiable, and reproducible with acceptable precision (<20%) and accuracy (within 80–120%). The extraction recovery, matrix effect, dilution integrity, and stability (under bench-top, freeze–thaw, post-preparative, and long-term conditions) in addition to the intra-assay (five different samples) and inter-assay (five different runs) precision and accuracy for each analyte were determined at the LLOQ and three different QC levels as described in the US FDA guidelines and in previous studies [11,17,18,19].

### 2.6. In Vivo Pharmacokinetic Study in Rats

The animal experiment protocols used in the present study were approved by the Pusan National University-Institutional Animal Care and Use Committee for scientific care and ethical procedures (Busan, Korea; approval date: 01/05/2018; approval number: PNU-2018-1848). Male Sprague–Dawley rats (8-week-old; approximately 250 g) were purchased from DBL Co., Ltd. (Incheon, Korea). The rats were acclimatized in a clean room of the Laboratory Animal Center of Pusan National University (Busan, Korea) for 1 week. Then, they were fasted for 12 h prior to the pharmacokinetic experiment and then anesthetized by intramuscular injection of Zoletil at a dose of 20 mg/kg [20,21]. The femoral vein and artery of the rats were cannulated with a polyethylene tube (BD Medical; Franklin Lakes, NJ, USA). At approximately 4–6 h after the surgery for cannulation, an intravenous dose of DUT (2.5 mg/kg) simultaneously with or without intravenous KET (20 mg/kg) or an oral dose of DUT (5 mg/kg) simultaneously with or without intravenous KET (20 mg/kg) was administered to the rats. Drugs were dissolved in a vehicle consisting of dimethyl sulfoxide, ethanol, and polyethylene glycol 400 at a ratio of 10:5:85 (*v/v/v*). Blood (approximately 200 μL) was collected in heparin pre-treated microcentrifuge tubes via the femoral artery at 0, 1, 5, 15, 30, 60, 120, 180, 240, 360, 480, and 1200 min after the intravenous dose and at 0, 5, 15, 30, 60, 120, 150, 180, 240, 360, 480, and 1200 min after the oral dose. Following centrifugation of the blood samples at 3000× *g* at 4 °C for 10 min [19], 100 μL aliquots of plasma were stored at −80 °C.

### 2.7. In Vitro Microsomal Metabolism, Protein Binding, and Blood Distribution Studies

In vitro metabolism studies in RLM and HLM were conducted as previously reported with slight modifications [21,22]. To investigate the NADPH-dependent metabolism (via phase I enzymes including CYPs) of DUT in RLM and HLM, the disappearance of DUT at various concentrations ranging from 1 to 100 μM in the presence of NADPH was determined in RLM and HLM. The substrate (DUT) concentration ([S]; μM) versus the initial metabolic rate (V; pmol/min/mg protein) data were interpreted based on the Michaelis–Menten kinetics, and relevant parameters were estimated by nonlinear regression analysis (GraphPad Prism ver. 5.01; GraphPad Software, San Diego, CA, USA) based on the following Michaelis–Menten equation:

$$V\;=\;\frac{V_{\max\;}\times \;[S]}{K_{m}\;+\;[S]}$$

where V_max_ and K_m_ are the maximal metabolic rate and Michaelis–Menten constant, respectively. The intrinsic metabolic clearance (CL_int_) was calculated by dividing V_max_ by K_m_. The unbound K_m_ (K_m,u_) was calculated as the K_m_ multiplied by the unbound fraction in microsomes (f_u,mic_). The unbound CL_int_ (CL_int,u_) was calculated as the CL_int_ divided by the f_u,mic_.

To investigate the metabolic interaction potential between DUT and KET, the disappearance of DUT (5 μM) in RLM and HLM was determined under the absence and presence of KET at various concentrations ranging from 1 to 5000 nM. A microsomal reaction mixture was prepared as follows (total volume: 0.2 mL): 1 mM NADPH, 10 mM MgCl_2_, substrate (DUT), and inhibitor (KET), RLM or HLM (1 mg/mL), and 50 mM phosphate buffer. At 0 and 90 min after starting the metabolic reaction, a 50 μL aliquot of microsomal incubation mixture was sampled and immediately transferred into a clean 1.5 mL microcentrifuge tube containing 100 μL cold ACN with an internal standard (celecoxib at 400 ng/mL) to stop the metabolic reaction. After vortex mixing and centrifugation at 15,000× *g* for 10 min, a 100 μL aliquot of the supernatant was stored at −80 °C. The unbound fractions of DUT in rat plasma, RLM, human plasma, and HLM were measured using a rapid equilibrium dialysis (RED) device (Thermo Fisher Scientific, Inc., Waltham, MA, USA) as described in a previous study [23]. Then, 1 mL plasma or RLM (1 mg/mL) was spiked with either DUT alone or DUT with KET, yielding their final concentrations of 5 μM. A 0.2 mL aliquot of the spiked plasma or liver microsomes was placed into the “sample” chamber, and a 0.35 mL isotonic phosphate buffered saline was placed into the adjacent “buffer” chamber. The fraction unbound was calculated as the ratio of the drug concentrations in the “buffer” chamber to those in the “sample” chamber. The blood-to-plasma concentration ratio (R_B_) of DUT was determined as described previously [24,25]. Briefly, a 1 mL fresh blood was spiked with either DUT alone or DUT with KET, yielding their final concentrations of 5 μM, and then incubated at 37 °C for 60 min. Plasma was prepared by the centrifugation of the blood sample at 2000× *g* for 5 min. The concentrations of DUT in the plasma sample were determined by the validated HPLC analysis.

### 2.8. Data Analysis

The half maximal inhibitory concentration (IC_50_) of KET for the inhibition of the DUT metabolism was determined by the nonlinear regression analysis (GraphPad Prism ver. 5.01; GraphPad Software Inc., San Diego, CA, USA) based on the following Hill equation:

$$y\;=\;\mathrm{Min}\;+\;\frac{\mathrm{Max}-\mathrm{Min}}{1\;+\;(\frac{x}{{\mathrm{IC}}_{50}}{)}^{-P}}$$

where Max and Min are the initial and final y value, respectively, and the exponent P represents the Hill coefficient. Peak plasma concentration (C_max_) and time to reach C_max_ (T_max_) were read directly from the observed data. Non-compartmental analysis was conducted to estimate pharmacokinetic parameters such as total area under plasma concentration versus time curve from time zero to time of last sampling (AUC_last_), total area under plasma concentration versus time curve from time zero to infinity (AUC_inf_), total plasma clearance (CL), volume of distribution at steady state (V_ss_), and terminal half-life (t_1/2_) using the NCA200 and 201 models of WinNonlin ver. 3.1 (Certara USA Inc., Princeton, NJ, USA).

### 2.9. Statistical Analysis

A *p*-value below 0.05 was regarded as statistically significant, which was estimated by *t*-test for comparison between two unpaired means. All data except T_max_ were expressed as mean ± standard deviation, while T_max_ was expressed as median (range). All values were rounded to three significant figures.

## 3. Results

### 3.1. Method Development and Optimization

**Figure 2:**
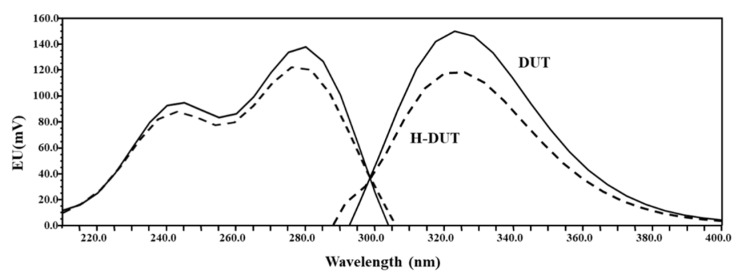
Fluorescence spectra of methanolic solution of DUT and H-DUT (10 μg/mL). The solid and dotted lines indicate the excitation and emission spectra, respectively.

Native fluorescence spectra of DUT and its metabolite H-DUT were measured using a spectrofluorometer. As shown in Figure 2, the fluorescence emission profiles of the two compounds were similar, exhibiting the maximum fluorescence intensity at excitation and emission wavelengths of 280 and 323 nm, respectively. The wavelength pair for the internal standard was set at 240 nm/380 nm (excitation/emission), which was optimized in our previous study [18]. The composition of mobile phase was optimized with respect to the pH and ACN content. The pH of the mobile phase exerted no discernible influence on the peak retention times of DUT and celecoxib, which are neutral compounds. Several trials with varying ACN contents showed that the gradient elution of mobile phase with ACN content ranging from 42% to 50% achieved acceptable separation and peak resolution. A few HPLC columns including a CAPCELL PAK C18 (250 × 4.6 mm, 5 μm; Shiseido Co., Tokyo, Japan) and ZORBAX HILIC Plus (100 × 4.6 mm, 3.5 μm; Agilent Technologies Inc., Santa Clara, CA, USA) were also tested. As a result, a Kinetex^®^ C8 column (250 × 4.6 mm, 5 μm; Phenomenex, Torrance, CA, USA) was found to offer better peak resolution than the other columns (data not shown). A solvent precipitation–reconstitution method was used for sample preparation owing to its simple procedure and low cost compared to liquid–liquid extraction and solid phase methods. Among several organic solvents such as methanol, ACN, trichloroacetic acid and their mixtures, deproteinization with ACN and subsequent centrifugation at 15,000× *g* in a relatively short precipitation time of 5 min offered acceptable matrix effect and peak resolution for DUT and H-DUT. Various fluorescent compounds including diclofenac, diflunisal, quinidine, and naproxen were tested, and celecoxib was found to exhibit good separation and suitable retention time.

### 3.2. Selectivity, Linearity, Sensitivity, Precision, and Accuracy

**Figure 3:**
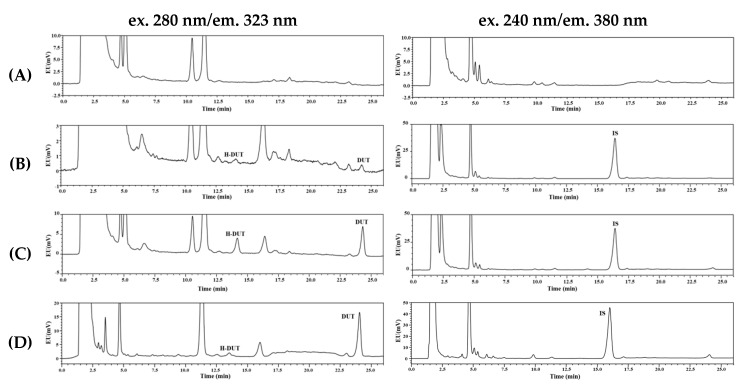
Representative chromatograms of DUT, H-DUT, and internal standard (IS) in rat plasma samples: (**A**) blank rat plasma; (**B**) blank rat plasma spiked with the analytes at the lower limit of quantification (LLOQ; 10 ng/mL); (**C**) blank rat plasma spiked with the analytes at the middle quantifiable concentration level (MQC; 150 ng/mL); (**D**) plasma sample collected at 30 min after the oral administration of DUT at a dose of 5 mg/kg in rats, where the calculated concentration of DUT was 276 ng/mL.

As shown in the typical HPLC chromatograms of blank rat plasma, blank rat plasma spiked with the analytes, and rat plasma sample obtained from intravenous pharmacokinetic study, no interfering endogenous substance was observed in the blank plasma at the retention times of DUT, H-DUT, and internal standard (Figure 3). The linear calibration curves (drug–internal standard peak area ratio plotted versus drug–internal standard concentration ratio) were obtained over the range from 10 to 1000 ng/mL. Representative fitted equations of the calibration curves are as follows:

DUT: *y* = 0.4144 × *x* − 0.0028 (*r*^2^ = 0.999)

H-DUT: *y* = 0.2267 × *x* + 0.0004 (*r*^2^ = 0.999)

where *x* is the ratio of nominal concentration of DUT or H-DUT to that of the internal standard and *y* is the ratio of peak area of DUT or H-DUT to that of the internal standard. The sensitivity of a bioanalytical method is defined by the LLOQ value [17], which was determined as 10 ng/mL for both DUT and H-DUT in this study. The intra- and inter-assay precision and accuracy were determined for the QC samples of DUT and H-DUT at four different concentration levels, i.e., LLOQ, LQC, MQC, and HQC. As shown in Table 1, the precision was 6.86% or less and the accuracy ranged from 97.2% to 108%.

### 3.3. Recovery, Matrix Effect, Dilution Integrity, and Stability

The recovery and matrix effect are listed in Table 2. The mean recoveries for DUT and H-DUT at the four QC levels ranged from 93.2% to 97.6% with CV values less than 4.2%. The mean matrix effect for DUT and H-DUT at the four QC levels ranged from 91.4% to 100% with CV values less than 5.7%. To confirm dilution integrity, the precision and accuracy of plasma control sample at 2000 ng/mL (2 times of the ULOQ) were determined to be 1.97% (precision) and 101% (accuracy), respectively, by conducting a 4-fold dilution. The bench-top, autosampler, freeze–thaw, and long-term stabilities of DUT and H-DUT were determined using QC samples (Table 3). The mean remaining fraction of DUT and H-DUT ranged from 95.1% to 108% with CV values less than 8.9%.

### 3.4. In Vivo Pharmacokinetic Drug Interaction Study in Rats

**Figure 4:**
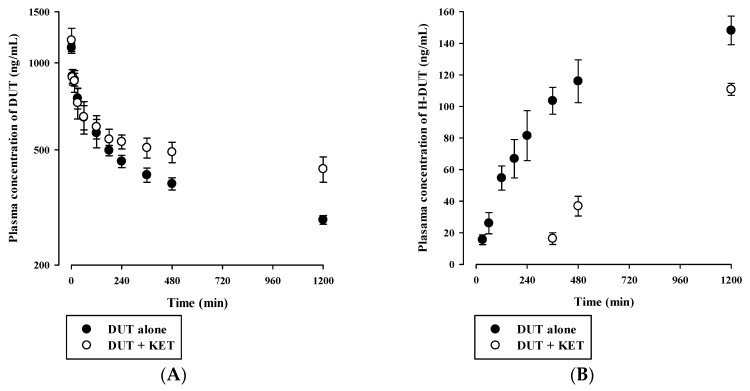
Plasma concentration versus time profiles of (**A**) DUT (closed circle) and (**B**) H-DUT (open circle) following the intravenous administration of DUT without or with intravenous KET in rats. The circles and vertical bars represent the means and standard deviations, respectively (*n* = 4).

**Figure 5:**
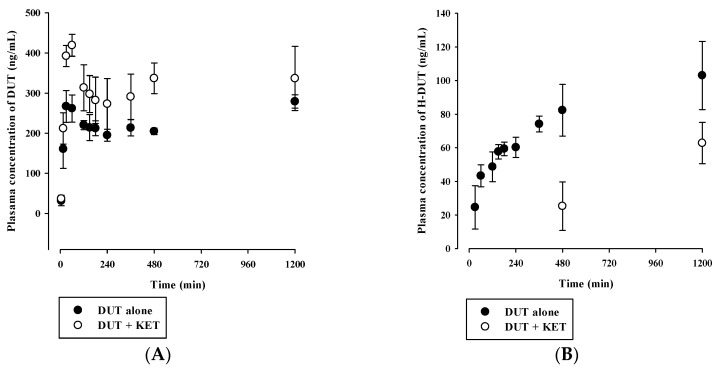
Plasma concentration versus time profiles of (**A**) DUT (closed circle) and (**B**) H-DUT (open circle) following the oral administration of DUT without or with oral KET in rats. The circles and vertical bars represent the means and standard deviations, respectively (*n* = 4).

Plasma concentration versus time profiles of DUT and H-DUT following intravenous and oral administration of DUT with or without KET in rats are shown in Figure 4 and Figure 5, and the relevant pharmacokinetic parameters are summarized in Table 4 and Table 5. In the intravenous study, the plasma concentration levels of DUT decreased in a multi-exponential manner, while those of H-DUT increased constantly during the entire period of blood collection (Figure 4). Thus, the AUC_inf_, t_1/2_, and F of H-DUT could not be determined in the oral study. The AUC_inf_, t_1/2_, and CL of DUT were significantly higher (*p* = 0.000449), higher (*p* = 0.000395), and lower (*p* = 0.0000467), respectively, and the AUC_last_ and C_max_ of H-DUT were significantly lower (*p* = 0.000194 and 0.0256, respectively) in the administration of DUT with KET than without KET. Consequently, the AUC ratio of H-DUT to DUT was significantly reduced (*p* = 0.0000804) by the concurrent administration of DUT and KET (Table 4). However, there was no significant difference in the V_ss_ between the two rat groups (*p* = 0.0921). In the oral study, the plasma concentration levels of DUT fluctuated with no discernible linear terminal phase, and those of H-DUT increased constantly during the entire period of blood collection (Figure 5). Thus, the AUC_inf_ and t_1/2_ of DUT and H-DUT could not be determined in the oral study. As shown in Table 5, the AUC_last_ and C_max_ of DUT and H-DUT were significantly higher (for DUT, *p* = 0.00870 and 0.000110, respectively; for H-DUT, *p* = 0.000381 and 0.0147, respectively) in the administration of DUT with KET than without KET. Consequently, the AUC ratio of H-DUT to DUT was significantly reduced (*p* = 0.000243) by the concurrent administration of DUT and KET. 

### 3.5. In Vitro Microsomal Metabolism, Protein Binding, and Blood Distribution Studies

**Figure 6:**
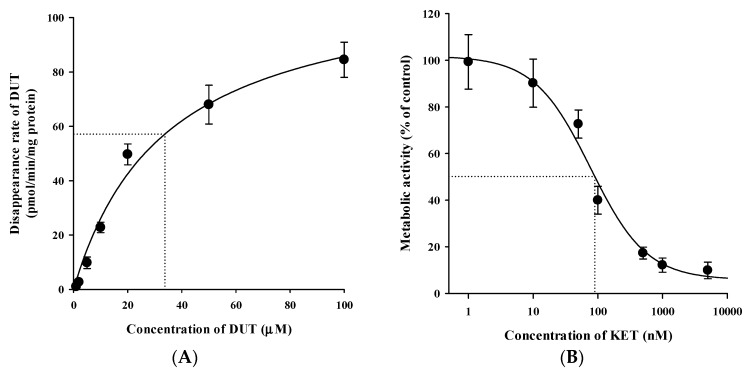
(**A**) Concentration dependence of the disappearance of DUT and (**B**) dose-response curves for the inhibitory effect of KET on the disappearance of DUT in rat liver microsomes (RLM). The closed circles and vertical bars represent the means and standard deviations, respectively (*n* = 5). The solid lines represent the fitted nonlinear regression curves.

**Figure 7:**
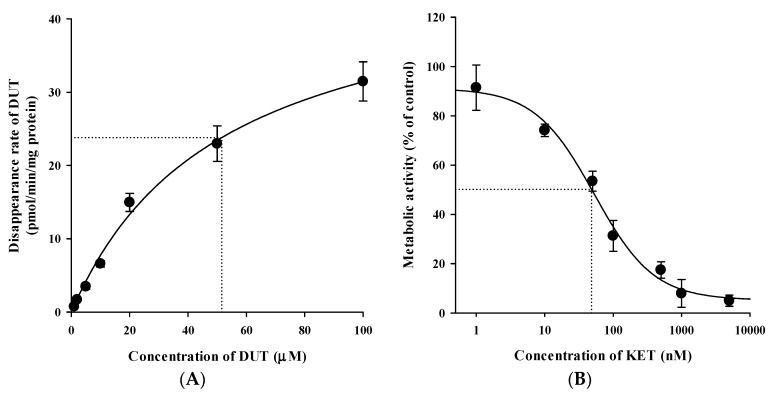
(**A**) Concentration dependence of the disappearance of DUT and (**B**) dose-response curves for the inhibitory effect of KET on the disappearance of DUT in HLM. The closed circles and vertical bars represent the means and standard deviations, respectively (*n* = 3). The solid lines represent the fitted nonlinear regression curves.

**Figure 8:**
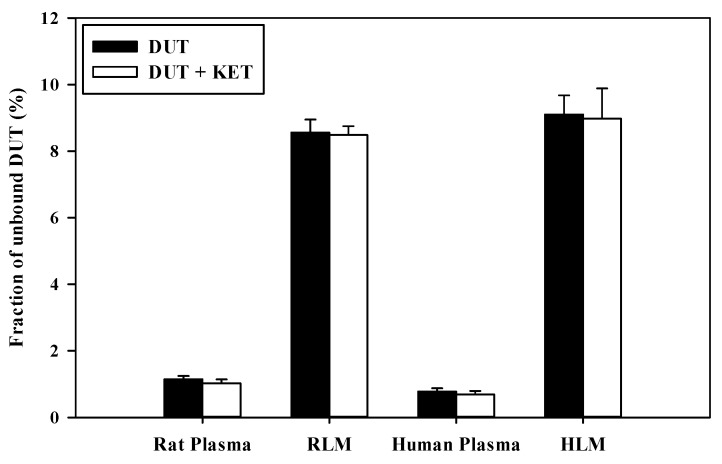
Fraction of unbound DUT in rat plasma, RLM, human plasma, and human liver microsomes (HLM) in the absence or presence of KET (*n* = 5).

Figure 6 and Figure 7 show the concentration dependence of DUT metabolism and the dose-response relationship for the inhibition of DUT metabolism by KET in RLM and HLM, respectively. As shown in Figure 6A and Figure 7A, the metabolic rate versus concentration profiles were well described by the Michaelis–Menten kinetics with a single saturation component in both RLM and HLM. The V_max_, K_m_, and CL_int_ of DUT were determined to be 114 ± 10 pmol/min/mg protein, 33.3 ± 3.6 μM, and 3.45 ± 0.37 μL/min/mg protein, respectively, in RLM, and 47.7 ± 6.3 pmol/min/mg protein, 51.6 ± 7.3 μM, and 0.928 ± 0.069 μL/min/mg protein, respectively, in HLM. The inhibition of the DUT metabolism by KET was well described by the sigmoidal Hill equation (Figure 6B and Figure 7B). Notably, KET significantly inhibited the metabolism of DUT with an IC_50_ of 77.3 ± 11.1 nM in RLM and 54.9 ± 15.6 nM in HLM. Additionally, the unbound fractions of DUT in rat plasma, RLM, human plasma, and HLM were determined to be 1.15% ± 0.10%, 8.56% ± 0.39%, 0.78% ± 0.10%, and 9.10% ± 0.57%, respectively. Based on these values, the K_m,u_ and CL_int,u_ of DUT were calculated to be 2.85 ± 0.31 μM and 40.3 ± 4.4 μL/min/mg protein, respectively, in RLM and 4.70 ± 0.67 μM and 10.2 ± 0.8 μL/min/mg protein, respectively, in HLM. In all the matrix tested, there were no significant differences in the fractions of unbound DUT between the cases with the absence and presence of KET (Figure 8). Similarly, the rat R_B_ of DUT was determined to be 0.712 ± 0.026, which was not significantly different from that in the presence of KET (0.692 ± 0.036; *p* = 0.448). 

## 4. Discussion

The present study was designed to investigate in vitro and in vivo metabolic drug interactions of DUT with KET in rats. To this end, a novel HPLC-FL method for the simultaneous determination of DUT and its major metabolite, H-DUT, was developed. Bioanalytical conditions were optimized to achieve sufficient sensitivity and separation of DUT and its metabolite from endogenous matrix components within a suitable run time. Several trials were performed to set up relevant factors including detector wavelength, mobile phase, stationary phase, sample preparation procedure, and internal standard. The parameters determined in the validation process were within generally accepted ranges, indicating that the HPLC-FL method proposed herein is precise, accurate, and reproducible. To the best of our knowledge, there have been no previous studies of an HPLC-FL method to quantify DUT and/or H-DUT in biological matrices. The steady-state serum DUT concentrations ranging from 16 to 78 ng/mL (median: 38 ng/mL) were reported in healthy humans who received 0.5 mg DUT once-daily for more than 6 months [6]. As the present bioanalytical method offered the LLOQ of 10 ng/mL using a relatively small volume of plasma sample (100 μL), it could become a promising alternative to LC-MS/MS methods for preclinical pharmacokinetic studies and clinical application after slight modification.

Although hepatic metabolism mediated by CYP3A4 and CYP3A5 is well recognized as a major elimination pathway of DUT in humans [6,7], little information has been available as to the hepatic CYPs involved in the metabolism of DUT in rats. The in vitro microsomal metabolism data presented in this study revealed that the metabolism of DUT was markedly reduced by KET, a potent inhibitor of CYP3A in rats and humans (Figure 6B and Figure 7B) [26,27]. This result indicates that CYP3A plays a significant role in the hepatic metabolism of DUT in rats and humans. The in vivo administered doses of DUT and KET were selected based on previous rat studies on these drugs [28,29,30,31]. In this study, the CL of DUT administered intravenously at a dose of 2.5 mg/kg was observed to be 2.19 ± 0.16 mL/min/kg in rats (Table 4). In a previous study [31] and our preliminary study, the CL of DUT at a dose of 1 mg/kg ranged from 2.17 to 4.3 mL/min/kg, which is comparable to the CL obtained in the present study. This suggests that DUT may follow linear pharmacokinetics at the doses tested. Considering the R_B_ of 0.712, the mean value of total blood clearance (CL_B_) is estimated to be 3.08 mL/min/kg (calculated as CL/R_B_). In our preliminary study, the renal excretion of DUT in the unchanged form was negligible (less than 1% of intravenously administered dose), which is consistent with previously reported human data [7]. Even if DUT is exclusively metabolized in the liver, the hepatic clearance (CL_H_) can be assumed to be equal to CL_B_, which is far less than the hepatic blood flow rate (Q_H_; 50–80 mL/min/kg in rats) [32,33]. This suggests that DUT may be regarded as a drug with a low hepatic extraction ratio of 0.039–0.062 (calculated as CL_H_/Q_H_). In addition, irregular and fluctuating plasma concentration versus time profiles were observed following the oral dosing of DUT (Figure 5A). This may be attributed to the variable gastrointestinal absorption and slow systemic clearance of DUT, which warrants further investigation.

Indeed, the AUC of intravenous and oral DUT in rats was significantly altered (1.6–2.1-fold increase) by KET administered via the same route as DUT (Table 4 and Table 5), which coincides well with the present in vitro metabolism data in RLM (Figure 6B). Furthermore, based on the FDA guidance [34], the magnitude of in vivo clinical drug–drug interactions between DUT and KET can be predicted from the in vitro metabolism data in HLM (Figure 7B). The ratio of the AUC of DUT in the presence and absence of KET is estimated to be 2.88 by the basic (simple static) model (for a detailed calculation process, see Table S2 in the Supplementary Information). This suggests that KET could act as an in vivo moderate inhibitor for DUT metabolism in clinical settings [35], which warrants further in vivo clinical investigation on the potential of pharmacokinetic interactions of DUT with KET and other chronic CYP3A inhibitors (e.g., ritonavir). Based on the well-stirred model, which is the most generally used hepatic clearance model, the CL_H_ of a drug with a low hepatic extraction ratio depends primarily on the CL_int_ and fu_B_ (calculated as fu_P_/R_B_) rather than the Q_H_ [36]. As the fu_P_ and R_B_ of DUT remained unaltered in the presence of KET (Figure 7), it is inferred that the CL_H_ of DUT is determined primarily by its CL_int_, which is representative of microsomal metabolism. This can be well supported by the consistency of metabolic drug interactions of DUT with KET between the present in vitro and in vivo studies.

Previous studies have suggested that systemic exposure to DUT, a CYP3A substrate, may be increased by co-administration with potent CYP3A inhibitors such as ritonavir, cimetidine, KET, and troleandomycin [6,37]. However, no clinical drug interaction studies have been yet conducted between DUT and these CYP3A inhibitors. To the best of our knowledge, the results presented herein are the first reported data on metabolism-based drug interactions between DUT and KET in rat and human in vitro and/or in vivo systems. Thus, this study could also shed light on the possibility of drug interactions of DUT with potent CYP3A inhibitors including azole antifungal agents such as KET, which requires further clinical investigation.

## 5. Conclusions

To our knowledge, this is the first study in which a simple, sensitive, and validated HPLC-FL method was developed for the simultaneous determination of DUT and its major metabolite H-DUT in rat plasma. The new HPLC-FL method offers several advantages including the simplicity of the sample preparation procedure, high extraction recovery, negligible matrix effect, and wide assay range that covers steady-state blood DUT concentrations observed in clinical settings. Its application to the drug interaction study between DUT and KET after their intravenous and oral administration revealed that the pharmacokinetics of DUT and H-DUT were significantly altered by the concurrent administration of KET. Furthermore, the in vitro microsomal metabolism, blood distribution, and protein-binding studies suggest that the altered pharmacokinetics of DUT could be primarily attributed to the inhibition of DUT metabolism by KET. The bioanalytical method described herein could serve as a promising alternative for preclinical pharmacokinetic studies and, by extension, clinical use after partial modification and validation.

## Figures and Tables

**Table 1:** Intra- and inter-assay precision and accuracy of DUT and H-DUT in rat plasma (*n* = 5).

Nominal Concentration (ng/mL)	Precision (%)		Accuracy (%)	
	Intra-Assay	Inter-Assay	Intra-Assay	Inter-Assay
DUT				
LLOQ (10)	1.67	5.83	108	104
LQC (30)	2.84	3.84	103	101
MQC (150)	1.27	2.43	101	100
HQC (750)	1.61	1.47	100	101
H-DUT				
LLOQ (10)	6.86	3.36	104	105
LQC (30)	6.48	3.91	99.0	97.2
MQC (150)	0.81	2.99	99.9	100
HQC (750)	1.00	1.42	102	100

**Table 2:** Recovery and matrix effect of DUT, H-DUT, and internal standard (IS) in rat plasma (*n* = 5).

Nominal Concentration (ng/mL)	Recovery (%)	Matrix Effect (%)
DUT		
LLOQ (10)	93.5 ± 4.1	97.5 ± 2.1
LQC (30)	93.3 ± 3.5	95.1 ± 2.1
MQC (150)	93.7 ± 1.1	96.9 ± 2.8
HQC (750)	95.4 ± 1.3	97.8 ± 1.5
H-DUT		
LLOQ (10)	93.2± 3.0	96.8 ± 3.1
LQC (30)	95.3 ± 4.2	94.0 ± 5.7
MQC (150)	96.6 ± 1.0	98.3 ± 2.1
HQC (750)	97.6 ± 1.8	100 ± 2
IS (celecoxib, 400)	96.8 ± 1.6	91.4 ± 0.3

**Table 3:** Stability (as percent drug remaining) of DUT in rat plasma (*n* = 5).

Nominal Concentration (ng/mL)	Bench-Top ^a^	Autosampler ^b^	Freeze‒Thaw ^c^	Long-Term ^d^
DUT				
LLOQ (10)	108 ± 1	103 ± 8	99.3 ± 4.7	105 ± 5
LQC (30)	105 ± 6	103 ± 1	99.7 ± 2.7	102 ± 4
MQC (150)	103 ± 1	104 ± 2	106 ± 2	100 ± 1
HQC (750)	102 ± 0	102 ± 2	100 ± 0	105 ±1
H-DUT				
LLOQ (10)	102 ± 4	103 ± 4	101 ± 7	96.1 ± 4.7
LQC (30)	104 ± 9	97.1 ± 0.8	99.0 ± 2.3	95.1 ± 3.7
MQC (150)	99.0 ± 1.4	103 ± 4	103 ± 3	98.7 ± 1.5
HQC (750)	101 ± 0	104 ± 1	101 ± 1	106 ± 1

^a^ Room temperature for 3 h; ^b^ 10 °C for 24 h in the autosampler; ^c^ Three freezing and thawing cycles; ^d^ −20 °C for 30 days.

**Table 4:** Pharmacokinetic parameters of DUT following its intravenous administration without or with intravenous KET in rats (*n* = 4).

Parameter	DUT alone	DUT with KET
DUT		
AUC_inf_ (μg·min/mL)	1148 ± 81	2506 ± 384 *
t_1/2_ (min)	1607 ± 196	3057 ± 359 *
CL (mL/min/kg)	2.19 ± 0.16	1.02 ± 0.16 *
V_ss_ (mL/kg)	4900 ± 326	4405 ± 373
H-DUT		
AUC_last_ (μg·min/mL)	128 ± 15	64.0 ± 4.1 *
C_max_ (ng/mL)	141 ± 17	116 ± 4 *
AUC_H-DUT_/AUC_DUT_	0.265 ± 0.031	0.107 ± 0.013 *

* Significantly different from the single group (*p* < 0.05).

**Table 5:** Pharmacokinetic parameters of DUT following its oral administration without or with oral KET in rats (*n* = 4).

Parameter	DUT alone	DUT with KET
DUT		
AUC_last_ (μg·min/mL)	275 ± 9	388 ± 58 *
C_max_ (ng/mL)	289 ± 10	419 ± 27 *
T_max_ (min)	60 (30–1200)	60
H-DUT		
AUC_last_ (μg·min/mL)	95.3 ± 14.2	37.8 ± 7.6 *
C_max_ (ng/mL)	103 ± 20	62.8 ± 12.3 *
AUC_H-DUT_/AUC_DUT_	0.348 ± 0.061	0.0981 ± 0.0198 *

* Significantly different from the single group (*p* < 0.05).

## Supplementary Materials

The following are available online at https://www.mdpi.com/1999-4923/11/12/673/s1, Table S1. Previous literatures on bioanalytical methods for DUT using HPLC system coupled with ultraviolet-visible (UV/Vis) and tandem mass (MS/MS) detector. Table S2. Parameters relevant to the estimation of R value.
