# Supplementary material for: In Vitro and In Vivo Assessment of Metabolic Drug Interaction Potential of Dutasteride with Ketoconazole
Source: Pharmaceutics. 2019 Dec 11;11(12):673. doi: 10.3390/pharmaceutics11120673 (PMC6956158; doi:10.3390/pharmaceutics11120673)
Supplement: Supplementary file 1 [file pharmaceutics-11-00673-s001.pdf]

pharmaceutics

Supplementary Materials: In Vitro and In Vivo Assessment of Metabolic Drug Interaction Potential of Dutasteride with Ketoconazole

Table S1. Previous literatures on bioanalytical methods for DUT using HPLC system coupled with ultraviolet-visible (UV/Vis) and tandem mass (MS/MS) detector.

Detector	Matrix	Sample Volume	Sample Preparation	Mobile Phase	Conc. Range	Validation	Ref.
LC-MS/MS	Human plasma	300 μL	LLE	Isocratic	0.1-25 ng/mL	Full	[1]
LC-MS/MS	Human plasma	500 μL	LLE	Gradient	0.1-25 ng/mL	Full	[2]
LC-MS/MS	Human plasma	500 μL	SPE	Gradient	1-100 ng/mL	Full	[3]
LC-MS/MS	Human plasma	500 μL	SPE	Isocratic	0.1-10 ng/mL	Full	[4]
LC-MS/MS	Human plasma	900 μL	LLE	Isocratic	0.5-50 ng/mL	Full	[5]
LC-MS/MS	Human plasma	1000 μL	LLE	Gradient	0.05-4 ng/mL	Full	[6]
LC-MS/MS	Human Serum	2000 μL	LLE	NR	0.025-2.5 ng/mL	Partial	[7]
LC-MS/MS	Human Urine	5000 μL	SPE	Gradient	NR	Partial	[8]
LC-MS/MS	Rat plasma	100 μL	LLE	Isocratic	1-500 ng/mL	Partial	[9]
LC-MS/MS	Rat plasma	100 μL	LLE	Isocratic	5-400 ng/mL	Partial	[10]
HPLC-UV	Rat blood	100 μL	Deproteinization	Gradient	NR	Partial	[11]

LLE: liquid–liquid extraction; SPE: solid-phase extraction; NR: not reported.

Table S2. Parameters relevant to the estimation of R value.

Parameter	Value	Description & Source
$f_{u,KET}$	0.01	Unbound fraction of KET in human plasma [12]
$f_{u,mic,KET}$	0.97	Unbound fraction of KET in HLM [12]
IC_{50} (μM)	0.0549	Figure 7B
S (μM)	5	Figure 7B
K_m (μM)	51.6	Figure 7A
K_i (μM)	0.0485	$f_{u,mic,KET} \times IC_{50} / (S/K_m + 1)$
$I_{max,u}$ (μM)	0.0913	[13]
R	2.88	$1 + I_{max,u} / K_i$

Based on the FDA guidance [14], the magnitude of in vivo clinical DDIs between DUT and KET was predicted using the basic (simple static) model as below, assuming that DUT is eliminated exclusively by hepatic CYP3A-mediated metabolism and KET acts as a competitive inhibitor for DUT metabolism [12].

$$R = 1 + I_{max,u}/K_i$$

where the R is the predicted ratio of the AUC of DUT in the presence and absence of KET, the $I_{max,u}$ is the maximum unbound plasma concentration of KET, and the K_i is the unbound inhibition constant of KET determined in vitro. Since IC_{50} only was determined in this study, it was converted to K_i using the following equation for competitive inhibition [15].

$$K_i = IC_{50}/(S/K_m + 1)$$

where the S is the fixed concentration of DUT, and the K_m is the Michaelis-Menten constant. The parameters relevant to the estimation of R value are listed in Table S2. Based on the FDA guidance

[16] and R value, it is plausible that KET acts as an in vivo moderate inhibitor for DUT metabolism in clinical setting, which warrants further in vivo clinical investigation on the potential of pharmacokinetic interactions of DUT with KET and other chronic CYP3A inhibitors (e.g., ritonavir).

References

1. Contractor, P.; Kurani, H.; Guttikar, S.; Shrivastav, P.S. Reliable and sensitive determination of dutasteride in human plasma by liquid chromatography-tandem mass spectrometry. *Biomed. Chromatogr.* **2013**, *27*, 1168–1176.
2. Ramakrishna, N.V.; Vishwottam, K.N.; Puran,S.; Koteshwara, M.; Manoj, S.; Santosh, M. Selective and rapid liquid chromatography-tandem mass spectrometry assay of dutasteride in human plasma. *J. Chromatogr. B.* **2004**, *809*, 117–124.
3. Upreti, R.; Naredo, G.; Faqehi, A.M.; Hughes, K.A.; Stewart, L.H.; Walker, B.R.; Homer, N.Z.; Andrew, R. Simultaneous pharmacokinetic and pharmacodynamic analysis of 5α-reductase inhibitors and androgens by liquid chromatography tandem mass spectrometry. *Talanta* **2015**, *131*, 728–735.
4. Gomes, N.; Pudage, A.; Joshi, S.; Vaidya, V.; Parekh, S.; Tamhankar, A. Rapid and Sensitive LC–MS–MS Method for the Simultaneous Estimation of Alfuzosin and Dutasteride in Human Plasma. *Chromatographia* **2009**, *69*, 9–18.
5. Agarwal, S.; Gowda, K.; Sarkar, A.K.; Ghosh, D.; Bhaumik, U.; Chattaraj, T.; Pal, T. Simultaneous determination of tamsulosin and dutasteride in human plasma by LC–MS–MS. *Chromatographia* **2008**, *67*, 893–903.
6. Prasaja, B.; Harahap, Y.; Lusthom, W.; Yumi, L.; Sofiana, A.; Sandra, M.; Safira, F.; Chilmi, U. Impact of Truncated Area on Point Estimate and Intra-Subject Variability in Bioequivalence of Dutasteride with Long Half-Life. *Drug Res.* **2018**, *68*, 238–240.
7. Fossler, M.J.; Collins, D.A.; Ino, H.; Sarai, N.; Ravindranath, R.; Bowen, C.L.; Burns, O. Evaluation of bioequivalence of five 0.1 mg dutasteride capsules compared to one 0.5 mg dutasteride capsule: a randomized study in healthy male volunteers. *J. Drug Assess.* **2015**, *4*, 24–29.
8. Brun, E.M.; Torres, A.; Ventura, R.; Puchades, R.; Maquieira, A. Enzyme-linked immunosorbent assays for doping control of 5α-reductase inhibitors finasteride and dutasteride. *Anal. Chim. Acta* **2010**, *671*, 70–79.
9. Beak, I.H.; Kim, M.S. Improved supersaturation and oral absorption of dutasteride by amorphous solid dispersions. *Chem. Pharm. Bull.* **2012**, *60*, 1468–1473.
10. Kang, M.J.; Cho, H.; Yeom, D.; Choi, Y.; Choi, Y. A Method to Monitor Dutasteride in Rat Plasma Using Liquid-Liquid Extraction and Multiple Reaction Monitoring: Comparisons and Validation. *Mass Spectrometry Letters* **2014**, *5*, 79–83.
11. Bramson, H.N.; Hermann, D.; Batchelor, K.W.; Lee, F.W.; James, M.K.; Frye, S.V. Unique preclinical characteristics of GG745, a potent dual inhibitor of 5AR. *J. Pharmacol. Exp. Ther.* **1997**, *282*, 1496–1502.
12. Peters, S.A.; Schroeder, P.E.; Giri, N.; Dolgos, H. Evaluation of the use of static and dynamic models to predict drug-drug interaction and its associated variability: impact on drug discovery and early development. *Drug Metab. Dispos.* **2012**, *40*, 1495–1507.
13. Tsunoda, S.M.; Velez, R.L.; von Moltke, L.L.; Greenblatt, D.J. Differentiation of intestinal and hepatic cytochrome P450 3A activity with use of midazolam as an in vivo probe: effect of ketoconazole. *Clin. Pharmacol. Ther.* **1999**, *66*, 461–471.
14. USA Food and Drug Administration. Guidance for industry: in vitro metabolism and transporter-mediated drug-drug interaction studies. 2017. Available online: https://www.fda.gov/media/108130/download (accessed on 28 November 2019)
15. Cer, R.Z.; Mudunuri, U.; Stephens, R.; Lebeda, F.J. IC50-to-Ki: a web-based tool for converting IC50 to Ki values for inhibitors of enzyme activity and ligand binding. *Nucleic Acids Res.* **2009**, *37*, W441–445.
16. USA Food and Drug Administration. Guidance for industry: clinical drug interaction studies-study design, data analysis, and clinical implications. 2017. Available online: https://www. fda. gov/downloads/drugs/guidances/ucm292362. pdf (accessed on 28 November 2019).
